# Identification of Flavin-Containing Monooxygenase 5 (FMO5) as a Regulator of Glucose Homeostasis and a Potential Sensor of Gut Bacteria

**DOI:** 10.1124/dmd.117.076612

**Published:** 2017-09

**Authors:** Flora Scott, Sandra G. Gonzalez Malagon, Brett A. O’Brien, Diede Fennema, Sunil Veeravalli, Clarissa R. Coveney, Ian R. Phillips, Elizabeth A. Shephard

**Affiliations:** Institute of Structural and Molecular Biology, University College London, London, United Kingdom (F.S., S.G.G.M., B.A.O., D.F., S.V., C.R.C., I.R.P., E.A.S.); and School of Biological and Chemical Sciences, Queen Mary University of London, London, United Kingdom (I.R.P.)

## Abstract

We have previously identified flavin-containing monooxygenase 5 (FMO5) as a regulator of metabolic aging. The aim of the present study was to investigate the role of FMO5 in glucose homeostasis and the impact of diet and gut flora on the phenotype of mice in which the *Fmo5* gene has been disrupted (*Fmo5^−^^/^^−^* mice). In comparison with wild-type (WT) counterparts, *Fmo5^−^^/^^−^* mice are resistant to age-related changes in glucose homeostasis and maintain the higher glucose tolerance and insulin sensitivity characteristic of young animals. When fed a high-fat diet, they are protected against weight gain and reduction of insulin sensitivity. The phenotype of *Fmo5^−^^/^^−^* mice is independent of diet and the gut microbiome and is determined solely by the host genotype. *Fmo5^−^^/^^−^* mice have metabolic characteristics similar to those of germ-free mice, indicating that FMO5 plays a role in sensing or responding to gut bacteria. In WT mice, FMO5 is present in the mucosal epithelium of the gastrointestinal tract where it is induced in response to a high-fat diet. In comparison with WT mice, *Fmo5^−^^/^^−^* mice have fewer colonic goblet cells, and they differ in the production of the colonic hormone resistin-like molecule *β*. *Fmo5^−^^/^^−^* mice have lower concentrations of tumor necrosis factor *α* in plasma and of complement component 3 in epididymal white adipose tissue, indicative of improved inflammatory tone. Our results implicate FMO5 as a regulator of body weight and of glucose disposal and insulin sensitivity and, thus, identify FMO5 as a potential novel therapeutic target for obesity and insulin resistance.

## Introduction

Flavin-containing monooxygenases (FMOs) (Enzyme Commission #1.14.13.8) of mammals play an important role in the NADPH-dependent oxidative metabolism of a wide array of foreign chemicals (Krueger and Williams, 2005). FMOs 1, 2, and 3 catalyze the *N*- and *S*-oxygenation of a structurally diverse range of xenobiotic agents, including therapeutic drugs, dietary components, and environmental pollutants (Krueger and Williams, 2005; Phillips et al., 2007). FMO5 differs markedly from FMOs 1, 2, and 3 with respect to its substrate specificities (Phillips and Shephard, 2017). Although FMO5 catalyzes the *N*-oxygenation of short-chain aliphatic primary amines such as *N*-octylamine (Overby et al., 1995) and the *S*-oxygenation of *S*-methyl-esonarimod, an active metabolite of the antirheumatic agent esonarimod (Ohmi et al., 2003; Zhang et al., 2007), it is apparently more efficient in catalyzing the oxygenation of a variety of carbonyl compounds, via a Baeyer-Villiger process (Fiorentini et al., 2016), including the anticancer drug E7016 (Lai et al., 2011) and the antibacterial agent MRX-I (Meng et al., 2015). FMOs 1, 2, and 3 (Siddens et al., 2014) and FMO5 (Fiorentini et al., 2016) can act also as NADPH oxidases, producing hydrogen peroxide.

In human and mouse, the tissue-specific pattern of expression of FMO5 is similar, unlike those of FMO1 or FMO3, which show distinct differences in hepatic expression between these species (Dolphin et al., 1991, 1996; Janmohamed et al., 2004). FMO5 is most highly expressed in liver (Overby et al., 1997; Zhang and Cashman, 2006) and skin (Janmohamed et al., 2001) and is moderately expressed in other tissues, including the small intestine (Hernandez et al., 2004; Zhang and Cashman, 2006; Fu et al., 2016).

Very few single-nucleotide polymorphisms (SNPs) have been identified in the coding region of the human *FMO5* gene, and none has been shown to influence enzyme activity (Furnes et al., 2003; Phillips et al., 2007). *FMO5* gene expression, however, is increased by several chemicals, including the hormones progesterone (Miller et al., 1997) and testosterone (Houseman et al., 2015) and the pregnane X receptor ligands hyperforin (Krusekopf and Roots, 2005) and rifampicin (Rae et al., 2001; Houseman et al., 2015). Activation of pregnane X receptor has been proposed as a factor contributing to type 2 diabetes (Hukkanen et al., 2014). Interindividual variation in the expression of FMO5 in human liver has been reported (Overby et al., 1997). Given the inducible nature of the gene, such variation is likely due to physiologic or environmental factors.

Through the use of knockout mouse lines, we have shown that, in addition to their involvement in the metabolism of foreign chemicals, two members of the FMO family, FMO1 and FMO5, play roles in endogenous metabolism. FMO1 has been identified as a regulator of energy homeostasis (Veeravalli et al., 2014), and FMO5 has been identified as a promoter of metabolic aging (Gonzalez Malagon et al., 2015). Homozygous knockout (*Fmo5^−/−^*) mice exhibit an age-related lean phenotype: in comparison with their wild-type (WT) counterparts, as they age they gain less weight, store less fat in white adipose tissue (WAT), have lower plasma concentrations of glucose and cholesterol, and enhanced whole-body energy expenditure, with no increase in physical activity (Gonzalez Malagon et al., 2015).

The present study extends our characterization of the metabolic phenotype of *Fmo5^−/−^* mice, focusing on the potential role of FMO5 in regulating glucose homeostasis and the effects on the phenotype of diet and gut flora. We show that *Fmo5^−/−^* mice are resistant to age-related changes in glucose homeostasis and are protected against weight gain and reduction of insulin sensitivity in response to a high-fat diet. The phenotype is independent of the gut microbiome, and our results implicate FMO5 as an intestinal microbial sensor.

## Materials and Methods

### 

#### Animal Maintenance.

*Fmo5^−/−^* mice used in this study were generated on the C57BL/6J background and back-crossed for eight generations, as described previously (Gonzalez Malagon et al., 2015). WT C57BL/6J mice were used as controls. Both mouse lines were bred and housed in the same room at University College London (UCL). All experiments were performed on male animals. Mice were given free access to water and fed ad libitum with a standard chow diet (Teklad Global 18% Protein Rodent Diet; Harlan Laboratories, Inc., Madison, WI) or, where specified, a high-fat diet (AIN-93M w/ 35% kcal fat; TestDiet, St. Louis, MO). Tissue and blood samples were collected between 9:30 AM and 12:00 PM (noon), unless stated otherwise. Animal procedures were carried out in accordance with the UK Animal Scientific Procedures Act and with local ethics committee approval (Animal Welfare and Ethical Review Body) and appropriate Home Office Licenses.

#### Plasma Analyses.

Blood was collected and plasma was isolated as described previously (Hough et al., 2002). Mice fed a high-fat diet and their standard chow–fed age-matched controls were starved overnight before blood withdrawal. The concentration of glucose in plasma was determined via an autoanalyzer at the Medical Research Council Harwell (Harwell, Oxfordshire, UK) Mammalian Genomics Unit and in whole blood through the use of a CONTOUR XT Meter (Bayer AG, Leverkusen, Germany). Commercially available enzyme-linked immunosorbent assay kits were used to determine plasma concentrations of insulin (Rat/Mouse Insulin ELISA; Millipore, Watford, Hertfordshire, UK) and TNF*α* (TNF*α* Mouse EIA Kit; Enzo, Exeter, Devon, UK). Plasma lipopolysaccharide was measured by Lonza Endotoxin Service Europe (Verviers, Belgium) using the Limulus Amebocyte Lysate Assay.

#### Intraperitoneal Glucose Tolerance and Insulin Sensitivity Tests.

Glucose tolerance tests (GTTs) and insulin sensitivity tests (ISTs) were performed as described previously (Heikkinen et al., 2007) in animals that had been fasted either overnight (15 hours) (GTT) or for 6 hours (IST). Fasting blood glucose concentrations were measured through the use of a CONTOUR XT Meter (Bayer AG). Animals were then injected intraperitoneally with glucose (2 g/kg b.wt.) (GTT) or insulin (0.25 IU/kg b.wt.) (IST). Blood glucose concentration was then measured 15, 30, 60, 90, and 120 minutes (GTT) or 15, 30, 45, 60, 75, and 90 minutes (IST) postinjection. For statistical analysis, area under the curve (AUC) or inverse AUC was calculated as appropriate (Heikkinen et al., 2007).

#### Intestinal Microbiota Analysis.

DNA was isolated from frozen fecal pellets through the use of the Isolate Fecal DNA Kit (Bioline, London, UK) according to the manufacturer recommendations, except for an additional 30 minutes of shaking after addition of lysis buffer. Bacterial 16S ribosomal RNA (rRNA) gene sequences were amplified using the universal 16S rRNA primer pair, forward 8FE (5′-AGAGTTTGATCCTGGCTCAG-3′) (Salzman et al., 2002) and reverse 1387r (5′-GGGCGGTGTGTACAAGGC-3′) (Marchesi et al., 1998), and Biotaq DNA polymerase (Bioline). Approximately 16 ng of fecal DNA and 5 pmol each of forward and reverse primers were used per 25-*μ*l reaction volume. Polymerase chain reaction conditions were 5 minutes at 95°C, followed by 30 cycles of 95°C for 1 minute, 51°C for 1 minute, and 72°C for 1 minute, and a final elongation at 72°C for 5 minutes. Amplification products were ligated into pGEM-T (Promega, Southampton, Hampshire, UK). Electrocompetent *Escherichia coli* JM109 cells were transformed with ligation products. Recombinant colonies were individually stabbed into agar-containing 96-well plates. Plasmid isolation and DNA sequencing were performed by LGC Genomics GmbH (Berlin, Germany). Sequences were identified by comparison with known 16S rRNA gene sequences in the Ribosomal Database Project (http://rdp.cme.msu.edu).

#### Antibiotic Treatment.

At 30 weeks of age, animals were given ampicillin (1 g/l) and neomycin (0.5 g/l) in their drinking water for 14 days. Antibiotic-containing water was replenished twice weekly, and animals were given free access to the standard chow diet.

#### Histology and Immunohistochemistry.

Sections of digestive tract were fixed in 10% (v/v) neutral buffered formalin (Leica Biosystems, Milton Keynes, Buckinghamshire, UK). Tissue samples were embedded in paraffin and sections stained with hematoxylin and eosin at the Medical Research Council Mary Lyon Centre (Harwell, Oxfordshire, UK). Immunohistochemistry of tissue sections was performed by UCL Advanced Diagnostics (London, UK). FMO5 was detected using a polyclonal antibody (16864-1-AP; ProteinTech, Manchester, UK), diluted 1:100 (Antibody Diluent Background Reducing; Dako, Ely, Cambridgeshire, UK). RELM*β* was detected using a polyclonal antibody (ab11429; Abcam, Cambridge, Cambridgeshire, UK), diluted 1:400 (Bond Primary Antibody Diluent; Leica Biosystems). Cell nuclei were counterstained with hematoxylin. Colonic sections were stained with Alcian Blue and counterstained with Neutral Red (UCL Advanced Diagnostics). Goblet cells were quantified as described previously (Mello et al., 2012).

#### Western Blot Analysis.

Fecal samples, colon contents, and liver and epididymal adipose tissues were homogenized using a Tissue Lyser II (Qiagen, Crawley, West Sussex, UK) in ice-cold Triton lysis buffer (1% Triton X-100, 140 mM NaCl, 10 mM Tris-HCl, pH 8, 1 mM EDTA, 1 mM phenylmethylsulfonyl fluoride) containing Halt Protease and Phosphatase Inhibitor Cocktail (Thermo Scientific, Loughborough, Leicestershire, UK). To ensure sufficient homogenization samples were agitated for an additional 15 minutes (liver and adipose tissue) or 40 minutes (fecal and colon samples) at 4°C. Homogenates were centrifuged at 12,000*g* for 20 minutes at 4°C. For detection of FMO5 in fecal samples, homogenates were concentrated by precipitation with trichloroacetic acid then resuspended in 200 mM Tris-HCl (pH 8.5), 10 mM EDTA, 6 M urea, 0.5% (w/v) SDS. Samples were adjusted to final concentrations of 50 mM Tris-HCl, pH 6.8, 10% (v/v) glycerol, 2% (w/v) SDS, 0.25% (w/v) bromophenol blue, and 100 mM dithiothreitol. Samples were then heated at 95°C for 5 minutes and analyzed by SDS-PAGE and western blotting. Proteins were detected by the use of antibodies against FMO5 (16864-1-AP; ProteinTech), β-actin (66009-1-Ig; Proteintech), RELM*β* (ab11429; Abcam), complement component 3 (C3) (204869; Millipore), and enolase (sc-15343; Santa Cruz Biotechnology, Heidelberg, Germany). For primary antibodies against FMO5, RELM*β*, and enolase, a secondary anti-rabbit antibody conjugated to horseradish peroxidase (HRP) [Donkey Anti-Rabbit IgG H+L (HRP); Abcam] was used. For β-actin, goat anti-mouse secondary antibody IR 680 (Alexa Fluor A21057; Life Technologies Ltd, Paisley, UK) was used. For C3, a secondary anti-goat antibody conjugated to HRP (Rabbit Anti-Goat IgG-HRP Conjugate; Alpha Diagnostic, San Antonio, TX) was used. Blots were incubated with secondary antibodies for 1 hour at room temperature before enhanced chemiluminescence detection, as described previously (Gonzalez Malagon et al., 2015), using an Odyssey Fc Dual-Mode Imaging System (LI-COR, Cambridge, Cambridgeshire, UK). For fecal and colon contents, to control for protein loading, the amount of protein loaded onto each well was assessed by scanning densitometry of the whole length of each lane using GelQuant.Net software, version 1.8.2 (BiochemLabSolutions; biochemlabsolutions.com). Individual protein signals on western blots were quantified using LI-COR Image Studio Lite, version 3.1.4.

#### Statistical Analysis.

Data are expressed as the mean ± S.E.M. Statistical significance was determined using an unpaired two-tailed *t* test and GraphPad Prism (version 6.0e; GraphPad Software, Inc., La Jolla, CA). The following symbols were used throughout all figures and text to indicate the significance value: *P < 0.05, **P < 0.01, ***P < 0.001, ****P < 0.0001.

## Results

### 

#### *Fmo5^−^****^/^****^−^* Mice Are Resistant to Age-Related Changes in Glucose Homeostasis and Insulin Sensitivity.

**Fig. 1.**
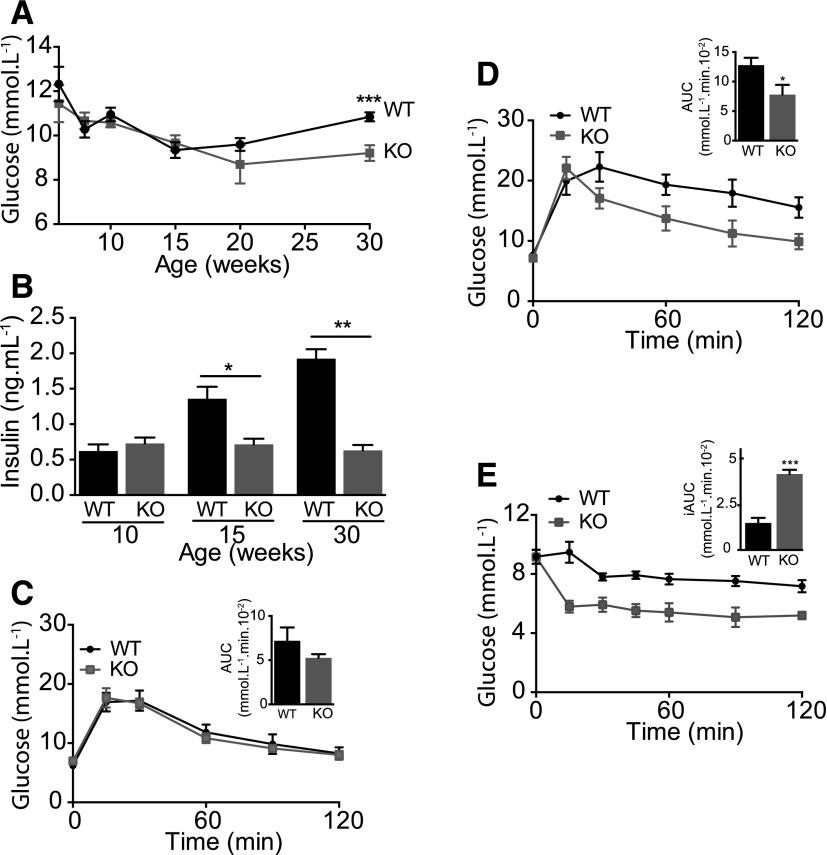
*Fmo5^−/−^* mice are resistant to age-related changes in glucose homeostasis. (A) Plasma glucose concentration of WT and *Fmo5^−/−^* [knockout (KO)] mice with age. 6 weeks: *n* = 4 (WT), *n* = 5 (KO); 8 weeks: *n* = 9 (WT), *n* = 11 (KO); 10 weeks: *n* = 21 (WT), *n* = 24 (KO); 15 weeks: *n* = 18 (WT), *n* = 23 (KO); 20 weeks: *n* = 10 (WT), *n* = 4 (KO); and 30 weeks: *n* = 18 (WT), *n* = 17 (KO). (B) Plasma insulin concentration of WT and *Fmo5^−/−^* (KO) mice with age: 10 and 15 weeks, *n* = 4; 30 weeks, *n* = 3. (C) GTT of 15-week-old WT and *Fmo5^−/−^* (KO) mice (*n* = 6). Insert shows the AUC. (D) GTT of 30-week-old WT and *Fmo5^−/−^* (KO) mice (*n* = 5). Insert shows the AUC. For GTT, mice were starved for 15 hours before the test. (E) IST of 10-week-old WT and *Fmo5^−/−^* (KO) mice that had been starved for 6 hours (*n* = 4). Insert shows inverse AUC (iAUC). Data are expressed as the mean ± SEM. *P < 0.05, **P < 0.01, ***P < 0.001.

The plasma concentration of glucose in 6-week-old *Fmo5^−/−^* mice was similar to that in WT mice (Fig. 1A). From 6 to 15 weeks of age it decreased, to the same extent, in both *Fmo5^−/−^* and WT mice. A similar decrease has been reported for mice as they age from immature to young adult (Bailey and Flatt, 1982). From 15 weeks of age, the plasma glucose concentration of WT mice, but not of *Fmo5^−/−^* mice, increased and by 30 weeks was 10.85 ± 0.20 mmol/l (*n* = 18) in WT mice and 9.21 ± 0.35 mmol/l (*n* = 17) in *Fmo5^−/−^* mice (*P* < 0.001, *Fmo5^−/−^* versus WT) (Fig. 1A). The results confirm and extend our previous findings on 10- and 30-week-old mice (Gonzalez Malagon et al., 2015) and indicate that the lower plasma concentration of glucose in *Fmo5^−/−^* mice compared with WT mice at 30 weeks of age is the result of a lack of increase, rather than a decrease, in plasma glucose of *Fmo5^−/−^* mice as they age from 15 to 30 weeks. At 15 and 30 weeks of age, both WT and *Fmo5^−/−^* mice responded to a 12-hour overnight withdrawal of food by decreasing their plasma glucose concentrations by ∼25% (data not shown).

At 10 weeks of age, the plasma concentration of insulin in *Fmo5^−/−^* and WT mice was the same (Fig. 1B). As mice aged, the plasma insulin concentration of WT mice increased but that of *Fmo5^−/−^* mice remained the same, and by 30 weeks of age the plasma insulin concentration of WT mice (1.92 ± 0.13 ng/ml; *n* = 3) was 3-fold more than that of *Fmo5^−/−^* mice (0.63 ± 0.08 ng/ml; *n* = 3; *P* < 0.01). Therefore, at 15 and 30 weeks of age, although the plasma concentration of glucose of *Fmo5^−/−^* mice was the same or lower than that of WT mice (Fig. 1A), *Fmo5^−/−^* mice required less insulin to maintain the concentration.

Glucose tolerances for *Fmo5^−/−^* and WT mice were the same at 15 weeks of age (Fig. 1C). By 30 weeks, *Fmo5^−/−^* mice, despite having lower plasma insulin concentrations than WT mice (Fig. 1B), had significantly higher glucose tolerance (Fig. 1D). The similarity in peak glucose concentration suggests no difference in pancreatic *β*-cell function between *Fmo5^−/−^* and WT mice, whereas the larger AUC of WT mice is indicative of greater whole-body insulin resistance. As early as 10 weeks of age, *Fmo5^−/−^* mice responded to insulin more quickly and to a greater extent than did WT mice (Fig. 1E), indicating that the former had greater basal insulin sensitivity. Our results demonstrate that *Fmo5^−/−^* mice are resistant to the age-related changes in glucose homeostasis and insulin sensitivity exhibited by their WT counterparts.

#### *Fmo5^−^****^/^****^−^* Mice Are Resistant to Weight Gain and Reduction of Insulin Sensitivity in Response to a High-Fat Diet.

**Fig. 2.**
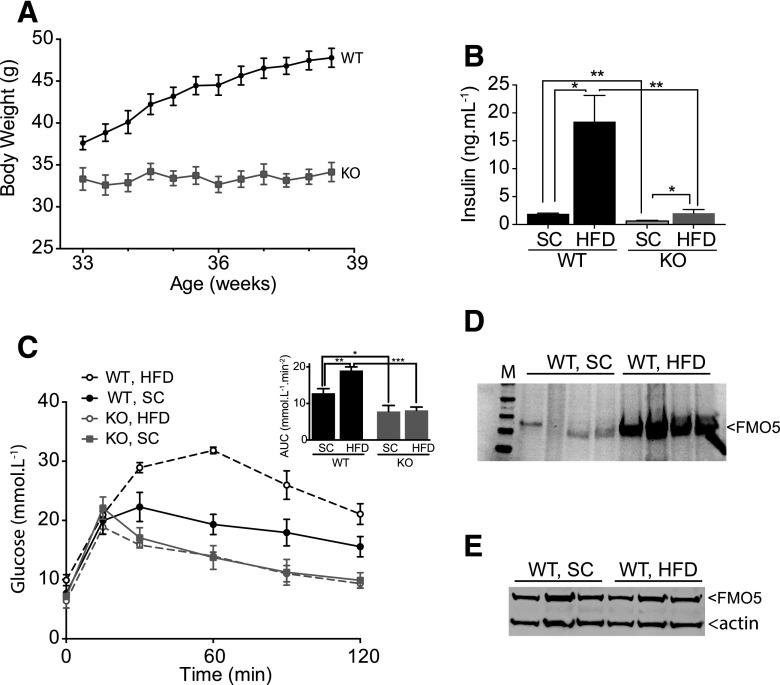
*Fmo5^−/−^* mice are resistant to weight gain and changes in glucose homeostasis in response to a high-fat diet. (A) Body weight of WT and *Fmo5^−/−^* (KO) mice fed a high-fat diet (WT, *n* = 7; KO, *n* = 6). (B) Plasma insulin concentrations of WT and KO mice fed standard chow (SC) (*n* = 3) or fed a high-fat diet (HFD) for 6 weeks (WT, *n* = 3; KO, *n* = 4). (C) GTT of WT and KO mice fed an SC diet (*n* = 5) or an HFD for 6 weeks (*n* = 4). Insert shows the AUC. (D) Western blot analysis of fecal proteins from WT mice fed an SC diet or an HFD for 6 weeks. Each lane represents proteins from a different mouse. M, molecular mass standards. The blot was incubated with an antibody against FMO5 and was developed as described in *Materials and Methods*. Protein loading was assessed as described in *Materials and Methods*. (E) Western blot analysis of liver proteins from WT mice fed an SC diet or an HFD for 6 weeks. Each lane represents proteins from a different mouse. The blot was incubated with an antibody against FMO5 or actin as the internal loading control. The blot was developed as described in *Materials and Methods*. Data in (A), (B), and (C) are expressed as the mean ± SEM. *P < 0.05, **P < 0.01, ***P < 0.001.

When fed a high-fat diet for a period of 6 weeks, 33-week-old WT mice increased in weight from 37.6 ± 0.7 to 46.2 ± 0.9 g (*n* = 7), a gain of 23% (Fig. 2A). In contrast, *Fmo5^−/−^* mice gained no weight while being fed the high-fat diet and by 39 weeks of age weighed 33.8 ± 1.0 g (*n* = 6), 27% less than WT mice (Fig. 2A). When fed a standard chow diet over the same period, neither WT nor *Fmo5^−/−^* mice of this age increased weight significantly (data not shown). When fed a standard chow diet, *Fmo5^−/−^* mice eat slightly more than WT mice (Gonzalez Malagon et al., 2015). In the case of the high-fat diet, owing to the consistency of the food pellets, which caused them to crumble when eaten, it was not possible to accurately measure food intake. However, the food hoppers of *Fmo5^−/−^* mice had to be replenished more frequently than those of WT mice, suggesting that, as is the case when being fed the standard chow diet, when fed a high-fat diet *Fmo5^−/−^* mice do not eat less than WT mice. Our results demonstrate that, in contrast to WT mice, *Fmo5^−/−^* mice are resistant to high-fat diet–induced weight gain and that this is unlikely to be a consequence of a reduced intake of food.

In response to a 12-hour overnight withdrawal of food, the plasma concentration of glucose in *Fmo5^−/−^* mice fed a high-fat diet was reduced to 6.98 ± 0.78 mmol/l (*n* = 6), which was similar to the concentration in fasted *Fmo5^−/−^* mice being fed a standard chow diet (7.28 ± 0.20 mmol/l; *n* = 23). However, WT mice responded less well to a 12-hour fast when being fed a high-fat diet than when being fed a standard chow diet, reducing plasma glucose concentrations to 9.61 ± 0.53 mmol/l (*n* = 7) and 7.97 ± 0.27 mmol/l (*n* = 20), respectively (*P* < 0.05).

The plasma concentration of insulin of WT mice fed a high-fat diet was 10-fold higher than that of WT mice fed a standard chow diet (Fig. 2B). In contrast, in *Fmo5^−/−^* mice the high-fat diet had a much smaller effect on plasma insulin, which was increased to a concentration similar to that of WT mice being fed a standard chow diet (Fig. 2B). The high-fat diet significantly reduced the glucose tolerance of WT mice but had no effect on that of *Fmo5^−/−^* mice (Fig. 2C). Our results demonstrate that, in comparison with WT mice, *Fmo5^−/−^* mice are resistant to changes in glucose homeostasis as a consequence of being fed a high-fat diet. Our finding that disruption of the *Fmo5* gene renders mice resistant to weight gain and a reduction of insulin sensitivity in response to a high-fat diet suggests that FMO5 is involved in mediating such dietary-induced physiologic effects.

#### FMO5 Is Induced in the Intestine in Response to a High-Fat Diet.

The amount of FMO5 present in fecal samples from WT mice was 6-fold higher in animals fed a high-fat diet than in samples from WT mice fed a standard chow diet (Fig. 2D), suggesting that intestinal expression of the protein was increased in response to a high-fat diet. However, this was not the case in liver (Fig. 2E), indicating that the high-fat diet–induced expression of FMO5 was tissue specific.

#### The Phenotype of *Fmo5^−^****^/^****^−^* Mice Is Independent of the Gut Microbiota.

**Fig. 3.**
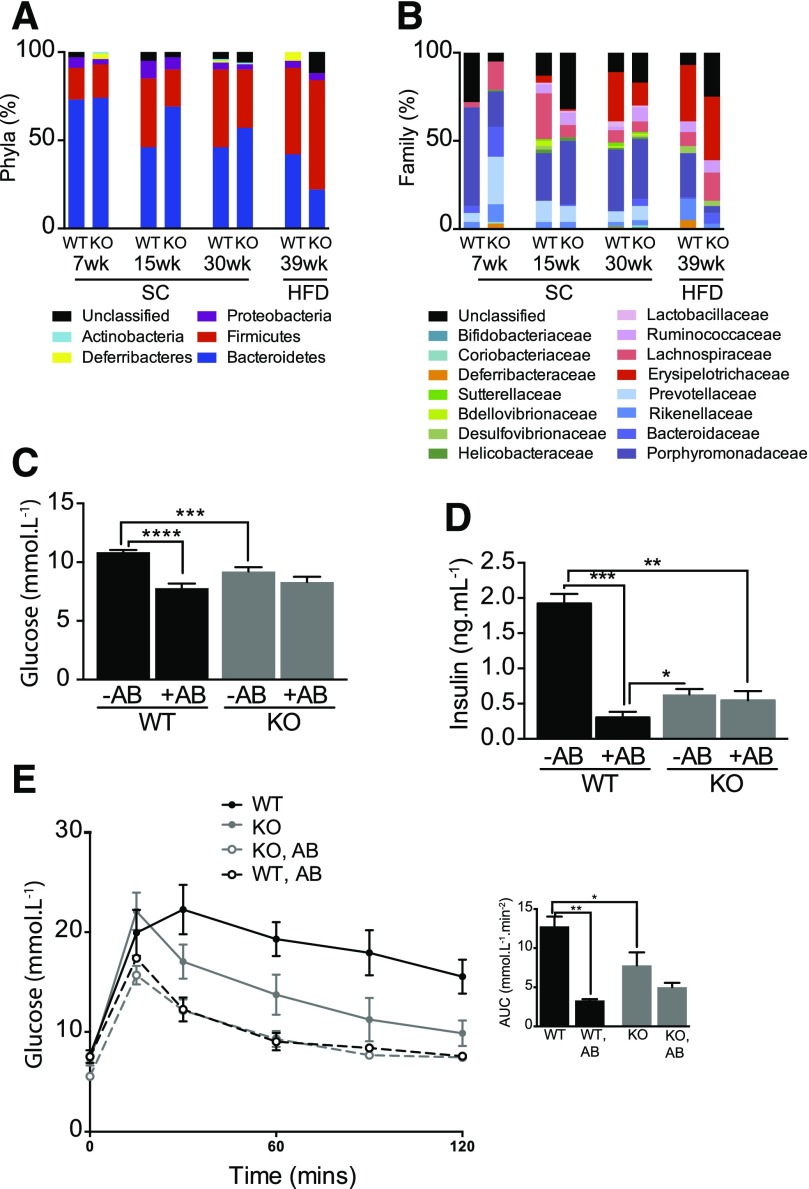
The phenotype of *Fmo5^−/−^* mice is independent of gut microbiota. Effect of age and diet on gut microbial phyla (A) and gut microbial families (B) of WT and *Fmo5^−/−^* (KO) mice. SC, mice were fed an SC diet; HFD, mice were fed an SC diet then a high-fat diet for 6 weeks. (C) Effect of antibiotic (AB) treatment on plasma concentrations of glucose of 30-week-old mice fed an SC diet (WT, *n* = 18; WT + AB, *n* = 4; KO, *n* = 17; KO + AB, *n* = 6). (D) Effect of AB treatment on plasma insulin concentration of 30-week-old mice fed an SC diet (*n* = 3 for each group). (E) Effect of antibiotic treatment on glucose tolerance of 30-week-old mice fed an SC diet. +AB, antibiotic treated (WT, *n* = 3; KO, *n* = 5); −AB, untreated (*n* = 5). Insert shows the AUC. The GTT was performed as described in *Materials and Methods*. Data in (C), (D), and (E) are expressed as the mean ± SEM. *P < 0.05, **P < 0.01, ***P < 0.001, ****P < 0.0001.

Five intestinal bacterial phyla were identified in WT and *Fmo5^−/−^* mice fed a standard chow diet: Actinobacteria, Proteobacteria, Deferribacteres, Firmicutes, and Bacteroidetes, of which the Firmicutes and Bacteroidetes were the most abundant (Fig. 3A). This is in agreement with previous studies of WT mice (Ley et al., 2005). At 7 weeks of age, the Bacteroidetes dominated, and the gut microbial community ratio of Bacteroidetes to Firmicutes was the same (4:1) in WT and *Fmo5^−/−^* mice (Fig. 3A). As WT mice aged, the relative abundance of Bacteroidetes decreased and that of Firmicutes increased, so that by 15 weeks of age the ratio of the phyla had decreased to ∼1 (Fig. 3A). A similar, but slower, change in the composition of gut flora occurred in the *Fmo5^−/−^* mice, with the Bacteroidetes-to-Firmicutes ratio decreasing to ∼3 at 15 weeks and ∼1.7 at 30 weeks of age (Fig. 3A). The relative abundance of Actinobacteria, Proteobacteria, and Deferribacteres was similar in WT and *Fmo5^−/−^* mice and did not change with age (Fig. 3A).

Although the ratio of Bacteroidetes to Firmicutes phyla was the same in 7-week-old WT and *Fmo5^−/−^* mice, the abundance of several bacterial families differed (Fig. 3B). For instance, Porphyromonadaceae were more prevalent in WT mice, and Prevotellaceae and Lachnospiraceae were more prevalent in *Fmo5^−/−^* animals. By 30 weeks of age, the Erysipelotrichaceae family, which was not detected in 7-week-old mice, was twice as abundant in WT as in *Fmo5^−/−^* animals. It is this family that contributed most to the difference in the ratio of Bacteroidetes to Firmicutes between 30-week-old WT and *Fmo5^−/−^* mice (Fig. 3B).

Six weeks of being fed a high-fat diet had little effect on the relative abundance of Bacteroidetes and Firmicutes in WT mice, decreasing the ratio of the phyla from 1.0 to 0.9, but had a marked effect in *Fmo5^−/−^* mice, decreasing the ratio from ∼1.7 to ∼0.4 (Fig. 3A). A lower ratio of Bacteroidetes to Firmicutes has been associated with an obesogenic state (Ley et al., 2005; Turnbaugh et al., 2009).

The relative abundance of several bacterial families changed in response to a high-fat diet (Fig. 3B). In the Bacteroidetes phylum, Rikenallaceae, reported to increase in response to high-fat feeding (Daniel et al., 2014), increased in WT but not in *Fmo5^−/−^* mice, whereas Porphyromonadaceae decreased, but to a much greater extent (from 33% to 4%) in *Fmo5^−/−^* mice than in WT mice. Within the Firmicutes phylum, the relative abundance of Lachnospiraceae increased in *Fmo5^−/−^* but not in WT mice. The most striking change was the almost 3-fold increase in Erysipelotrichaceae in *Fmo5^−/−^* mice. While being fed a high-fat diet, this family was the most abundant, comprising about a third of the total gut microbiota in both WT and *Fmo5^−/−^* mice. Erysipelotrichaceae have been reported to increase with diet-induced obesity (Kaakoush, 2015). The marked increase in the abundance of this family in *Fmo5^−/−^* mice fed a high-fat diet was not, however, accompanied by an increase in weight (Fig. 2A). The results indicate that in the *Fmo5^−/−^* mice the composition of gut microbiota changes in response to diet independently of an obese phenotype and that the phenotype of *Fmo5^−/−^* mice is not influenced by diet or gut flora.

Treatment with antibiotics for 2 weeks had a marked effect on WT mice, reducing the plasma concentration of glucose (Fig. 3C) and of insulin (Fig. 3D), but had no significant effect on *Fmo5^−/−^* mice (Fig. 3, C and D). Antibiotic treatment also significantly improved the glucose tolerance of WT mice (Fig. 3E). In the case of *Fmo5^−/−^* mice, although at initial time points in the GTT glucose concentrations were lower in antibiotic-treated mice, the AUC was not significantly different (Fig. 3E), indicating that antibiotic treatment did not significantly affect the glucose tolerance of the *Fmo5^−/−^* mice. Plasma glucose, plasma insulin, and glucose tolerances of antibiotic-treated WT mice were similar to those of non–antibiotic-treated *Fmo5^−/−^* mice (Fig. 3). Therefore, these metabolic parameters were profoundly influenced by gut flora in WT mice, but not in *Fmo5^−/−^* mice, indicating that the phenotype of *Fmo5^−/−^* mice is independent of the gut microbiota and is determined solely by host genotype.

#### FMO5 Is Expressed throughout the Murine Digestive Tract.

**Fig. 4.**
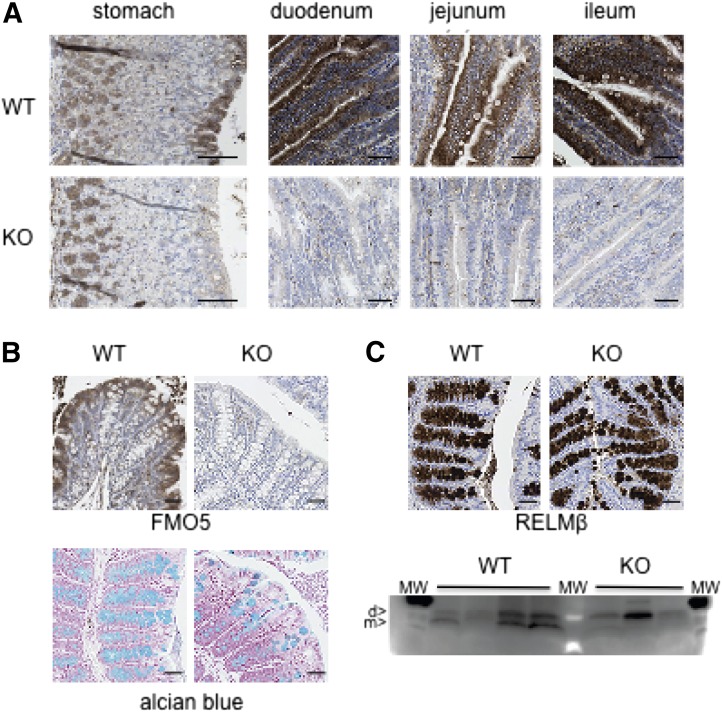
Expression of FMO5 and RELM*β* in mouse intestine. (A) Immunohistochemical analysis of sections of stomach and small intestine of 30-week-old mice. Sections were incubated with an antibody against FMO5. Top panels, WT mice; bottom panels, *Fmo5^−/−^* (KO) mice. Scale bar, 100 *μ*m. (B) Immunohistochemical analysis of sections of colons from 30-week-old mice. (Top panels) Sections incubated with antibodies against FMO5. (Bottom panels) Alcian blue staining of sections of colons from 30-week-old mice. Left-hand panels, WT mice; right-hand panels, KO mice. Scale bar, 100 *μ*m. (C) Immunohistochemical analysis of sections of colons of 30-week-old mice. (Top panels) Sections incubated with antibodies against RELM*β*. (Bottom panel) Western blot analysis of colonic content proteins from four WT and three KO mice. d, dimer; m, monomer; MW, molecular weight standards. Protein loading was assessed, and the blot was developed as described in *Materials and Methods*.

To determine whether FMO5 is expressed in the gut, sections of the digestive tract of WT mice were analyzed by immunohistochemistry (Fig. 4). Sections from the gut of *Fmo5^−/−^* mice were used as a negative control. The results revealed that FMO5 was present in the stomach, duodenum, jejunum, ileum (Fig. 4A), and colon (Fig. 4B). In each of these tissues, FMO5 was localized to the columnar epithelium of the mucosa, at the luminal surface. In the stomach, a signal associated with the lamina propria, at the base of the mucosa, was also observed in sections from *Fmo5^−/−^* mice, indicating that it was due to nonspecific binding of antibody. Analysis of sections of small intestine (Fig. 4A) and colon (Fig. 4B) of *Fmo5^−/−^* mice resulted in no signal, indicating that signals obtained from WT mice were specific for FMO5. Analysis of intestinal “Swiss rolls” confirmed that FMO5 was expressed throughout the length of both the small intestine and colon in WT mice (data not shown).

In both the small intestine (Fig. 4A) and large intestine (Fig. 4B), FMO5 was present throughout the columnar epithelium, including within goblet cells, which are distinguished from other epithelial cells by the presence of a visible secretory granule. FMO5 was not present in the secretory granule itself but was concentrated in the cytoplasm at the periphery of granules (Fig. 4, A and B). In the colon, the abundance of FMO5 increased from the base to the luminal opening of crypts (Fig. 4B), in parallel with the maturation of secretory granules.

The staining of transverse sections of stomach, small intestine and colon with hematoxylin and eosin showed no difference between WT and *Fmo5^−/−^* mice in the mucosal thickness or structure of these tissues (data not shown). Thus, although FMO5 was expressed throughout the mucosal epithelium of the murine gastrointestinal tract, in *Fmo5^−/−^* mice its absence did not affect the gross morphology of the gut.

#### *Fmo5^−^****^/^****^−^* Mice Have Fewer Colonic Goblet Cells.

Although there was no difference in colonic crypt length between WT mice (151.7 ± 3.6 *μ*m; *n* = 57) and *Fmo5^−/−^* mice (162.1 ± 5.0 *μ*m; *n* = 54), the number of goblet cells per crypt was significantly lower in *Fmo5^−/−^* mice (15.4 ± 0.7; *n* = 54) than in WT mice (18.5 ± 0.8; *n* = 57; *P* < 0.01) (Fig. 4B). Because *Fmo5^−/−^* mice have fewer goblet cells, we examined whether this difference influenced the expression of RELM*β*, a hormonally active protein present in goblet cell secretory granules, whose expression is confined to the colon (He et al., 2003). In WT mice, RELM*β* was localized to the crypts of the colonic mucosa, where it was concentrated in the secretory granules of goblet cells (Fig. 4C). As was the case for FMO5 (Fig. 4B), a gradient of expression of RELM*β*, increasing from the base of the crypt to the luminal opening, was observed, which is in agreement with the findings of a previous report (He et al., 2003). A similar pattern of RELM*β* expression was observed in the colon of *Fmo5^−/−^* mice (Fig. 4C), indicating that the disruption of the *Fmo5* gene had no effect on the localization of RELM*β*.

Analysis of proteins extracted from the luminal contents of the colon (Fig. 4C) identified an ∼17 kDa dimeric form of RELM*β* (He et al., 2003) in both WT and *Fmo5^−/−^* mice. However, in WT mice an additional band, migrating at ∼8 kDa, corresponding to the monomeric form of RELM*β* (He et al., 2003), was detected. Our results indicate that although *Fmo5^−/−^* mice have fewer colonic goblet cells, disruption of the *Fmo5* gene does not affect the expression of RELM*β* but may affect the production of the monomeric form of the protein in the colon. The expression of RELM*β* was markedly reduced by antibiotic treatment (data not shown), as previously reported (Hildebrandt et al., 2009).

#### *Fmo5^−^****^/^****^−^* Mice Have Lower Amounts of Inflammatory Markers.

**Fig. 5.**
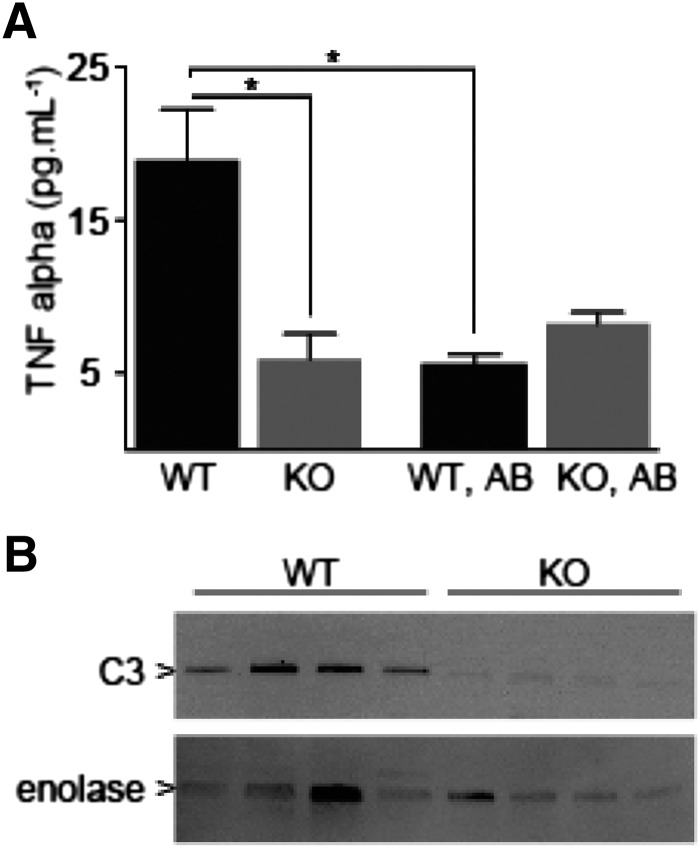
*Fmo5^−/−^* mice have lower amounts of inflammatory markers. (A) Plasma concentration of TNF*α* of 30-week-old WT and *Fmo5^−/−^* (KO) mice, either untreated (WT, *n* = 5; KO, *n* = 4) or treated with antibiotics (WT, AB; KO, AB) (*n* = 3). Data are expressed as the mean ± SEM. *P < 0.05. (B) Western blot analysis of proteins of WAT of 10-week-old WT and KO mice. The blot was incubated with antibodies against C3 or enolase, as a loading control. The blot was developed as described in *Materials and Methods*.

In view of the potential effect of *Fmo5* on the production of the monomeric form of RELM*β*, we measured markers of inflammation because, in addition to acting as a mucosecretagogue, RELM*β* is also regarded as an inflammatory mediator (Hogan et al., 2006; McVay et al., 2006; Krimi et al., 2008). The plasma concentration of the inflammatory cytokine TNF*α* was ∼70% lower in *Fmo5^−/−^* mice than in WT mice (Fig. 5A). Antibiotic treatment decreased the plasma concentration of TNF*α* in WT mice to a level similar to that in untreated *Fmo5^−/−^* mice but had no effect on plasma TNF*α* levels in *Fmo5^−/−^* mice (Fig. 5A). As was the case with plasma TNF*α*, the amount of C3, a visceral adipose inflammatory marker (Hertle et al., 2014), was lower in epididymal adipose tissue of *Fmo5^−/−^* mice than in that of WT mice (Fig. 5B). The plasma concentration of lipopolysaccharide in *Fmo5^−/−^* mice (3.34 ± 0.23 enzyme units/ml; *n* = 3) was the same as that in WT mice (3.11 ± 0.06 enzyme units/ml; *n* = 3), indicating that the disruption of the *Fmo5* gene resulted in no change in intestinal barrier integrity.

## Discussion

As WT C57BL/6J mice aged, they exhibited increased plasma concentrations of glucose and insulin and reduced glucose tolerance and insulin sensitivity. In contrast, mice in which the *Fmo5* gene had been disrupted, *Fmo5^−/−^* mice, were resistant to these changes in glucose homeostasis and maintained the higher glucose tolerance and insulin sensitivity that is characteristic of young animals. In response to a high-fat diet, WT mice increased weight, became hyperinsulinemic, and displayed markedly reduced glucose tolerance, which are indicative of increased whole-body insulin resistance. Again, *Fmo5^−/−^* mice were resistant to these changes, indicating that their metabolic phenotype is independent of diet. Our results demonstrate that disruption of the *Fmo5* gene protects mice against diet-induced obesity and the potentially deleterious effects on glucose homeostasis of both age and the consumption of high levels of fat and indicate that FMO5 plays a role in the regulation of body weight and of glucose disposal and insulin sensitivity.

Other mouse knockout lines are resistant to high-fat diet–induced weight gain, but in most cases, in contrast to *Fmo5*, the gene disrupted encodes a protein with a known role in fat metabolism or its regulation, for example, protein-tyrosine phosphatase 1B (Klaman et al., 2000), acetyl-CoA carboxylase 2 (Abu-Elheiga et al., 2012), and Cidea (Zhou et al., 2003). However, one example in which the product of the disrupted gene is not involved in fat metabolism or its regulation is that of RELM*β* (Hildebrandt et al., 2009).

Although the composition of the gut microbiota of *Fmo5^−/−^* mice changed with age and, in particular, in response to a high-fat diet to a composition associated with an obesogenic state, these changes had no effect on the metabolic phenotype of the mice. Disruption of a single gene, *Fmo5*, had a similar effect on plasma glucose, plasma insulin, and glucose tolerance as did antibiotic-mediated removal of gut microbiota from WT mice. Indeed, *Fmo5^−/−^* mice display metabolic characteristics associated with mice raised in the absence of microorganisms, that is, germ free, which, in comparison with conventionally reared mice, weigh less, store less fat, have lower plasma glucose and insulin concentrations, and greater insulin sensitivity and glucose tolerance (Backhed et al., 2007; Rabot et al., 2010). The similarities with germ-free mice suggest that in *Fmo5^−/−^* mice gut flora are “invisible” to the host and that FMO5 has a role in sensing or responding to gut bacteria.

Consistent with the proposed involvement of FMO5 in mediating the host response to gut flora is our finding that the protein is expressed in the epithelial lining of the gastrointestinal tract. The concentration of FMO5 at the periphery of secretory granules within goblet cells of the colon and the increase in the abundance of the protein as the cells matured suggests a possible role for FMO5 in events associated with the release or processing of granular components. One such component is RELM*β*. *Fmo5^−/−^* mice share some characteristics of a RELM*β* knockout mouse (Hildebrandt et al., 2009): both are protected from diet-induced obesity, and that protection is independent of diet-induced changes in intestinal microbiota. In addition, the expression of RELM*β*, which is confined to the colon (He et al., 2003), is induced in response to a high-fat diet (Hildebrandt et al., 2009), as was the case for intestinal FMO5 (Fig. 2D).

Our results indicate that the absence of FMO5 does not affect the expression of RELM*β* but may affect production in the colon of the monomeric form of the hormone. Studies of resistin, which is closely related to RELM*β*, indicate that the monomer is the active form of the hormone (Patel et al., 2004). If this is the case for RELM*β*, our results indicate that FMO5 may facilitate production of the active monomeric form of the hormone in the colon. Given the phenotypic similarities between *Fmo5^−/−^* and *RELMβ^−/−^* mice, it is possible that these result, in part, from altered production of the RELM*β* monomer in the *Fmo5^−/−^* mice.

Reduction in the plasma concentration of the inflammatory cytokine TNF*α* as a consequence of disruption of the *Fmo5* gene indicates that FMO5 has a deleterious effect on systemic inflammatory tone. RELM*β* has been identified as a promoter of TNF*α* synthesis and release from macrophages (McVay et al., 2006). Consequently, the effect of FMO5 on inflammatory tone might be mediated by its influence on the production of monomeric RELM*β*.

In addition to its effects on inflammation, TNF*α* has detrimental effects on insulin sensitivity (Kwon and Pessin, 2013), and a TNF*α* knockout mouse is protected against obesity-induced glucose intolerance and hyperinsulinemia (Uysal et al., 1997). Thus, our data suggest that the enhanced whole-body insulin sensitivity of *Fmo5^−/−^* mice results, in part, from the reduced expression of TNF*α*. The lower amount of C3 in epididymal WAT of *Fmo5^−/−^* mice is consistent with the lower plasma concentration in these animals of TNF*α*, which plays a role in regulating the expression of C3 (Cianflone et al., 2003). C3 is associated with the development of inflammation-linked metabolic disorders and reduces insulin function (Engström et al., 2005; Samaras et al., 2010; Hertle et al., 2014). Our findings suggest that reduced expression of C3 in WAT, a consequence of reduced circulating levels of TNF*α*, would contribute to the enhanced whole-body insulin sensitivity of *Fmo5^−/−^* mice. The proposed indirect effect on WAT of the disruption of the *Fmo5* gene is consistent with the lack of FMO5 expression in this tissue.

Consistent with its role as a regulator of endogenous metabolic processes, *FMO5* displays little genetic variation: within the coding region only two nonsynonymous SNPs have been identified, both of which are rare and occur only in individuals of recent African descent (Furnes et al., 2003; Phillips et al., 2007). FMO1, identified as a regulator of energy homeostasis (Veeravalli et al., 2014), also displays little genetic variation (Furnes et al., 2003; Phillips et al., 2007). This is in marked contrast to *FMO2* and *FMO3*, which are highly polymorphic (Phillips et al., 2007).

An SNP (rs7541245) within an intron of the *FMO5* gene has been reported to be marginally significantly associated with a decrease in glycemic response to metformin (Breitenstein et al., 2015). However, it is not known whether metformin can be metabolized by FMO5 and the effect of the SNP on expression or activity of FMO5 has not been established. There is an increasing appreciation of the role of the gut microbiome in metformin response (McCreight et al., 2016). Thus, if FMO5 does affect metformin response, our data suggest that it may do so via modulation of a host-gut microbiome interaction.

Our results have potential implications for humans. The effect that FMO5 has on the composition of the gut microbiome suggests that interindividual differences in the expression of intestinal FMO5 in humans would alter the gut microbiota and, thus, have potential effects on the efficacy of orally administered drugs that are metabolized by gut bacteria.

In addition, our results indicate that interindividual variation in the expression of *FMO5* (Overby et al., 1997) may contribute to differences in weight gain and insulin sensitivity and that the induction of FMO5 by some therapeutic agents (Miller et al., 1997; Rae et al., 2001; Krusekopf and Roots, 2005) may adversely affect the metabolic health of patients. Thus, FMO5 is identified as a potential novel therapeutic target for obesity and insulin resistance. Although the lack of FMO5 apparently promotes a “healthy” metabolic profile, its presence may have conferred an evolutionary advantage, particularly when food supplies were limited, by contributing to the ability of mammals, including humans, to deposit fat and increase weight.

## Abbreviations

AUC: area under the curve
C3: complement component 3
FMO: flavin-containing monooxygenase
GTT: glucose tolerance test
HRP: horseradish peroxidase
IST: insulin sensitivity test
RELM*β*: resistin-like molecule *β*
rRNA: ribosomal RNA
SNP: single-nucleotide polymorphism
TNF*α*: tumor necrosis factor *α*
UCL: University College London
WAT: white adipose tissue
WT: wild type
